# 5-Chloro-1-phenyl-1*H*-tetra­zole

**DOI:** 10.1107/S1600536811021210

**Published:** 2011-06-11

**Authors:** Xiu Guang Wang, Ying Wang

**Affiliations:** a Tianjin Key Laboratory of Structure and Performance for Functional Molecules, Tianjin Normal University, Tianjin 300387, People’s Republic of China

## Abstract

The tetra­zole and phenyl rings of the title compound, C_7_H_5_ClN_4_, form a dihedral angle 64.5°.

## Related literature

For the ferroelectric properties of tetra­zole derivatives, see: Sengupta & Mukherjee (2010 ▶). For their magnetic properties, see: Grunert *et al.* (2004 ▶); Van Koningsbruggen *et al.* (2000 ▶). For their luminescent properties, see: Wang *et al.* (2005 ▶).
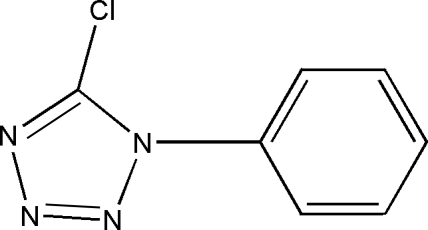


## Experimental

### 

#### Crystal data

C_7_H_5_ClN_4_
*M* *_r_* = 180.60
Monoclinic, 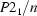

*a* = 7.0428 (7) Å
*b* = 6.4150 (6) Å
*c* = 17.5804 (18) Å
β = 96.160 (2)°
*V* = 789.69 (13) Å^3^
*Z* = 4
Mo *K*α radiation
μ = 0.43 mm^−1^
*T* = 296 K
0.15 × 0.14 × 0.13 mm

#### Data collection

Bruker SMART CCD area-detector diffractometer
Absorption correction: multi-scan (*SADABS*; Sheldrick, 1998 ▶) *T*_min_ = 0.939, *T*_max_ = 0.947
3879 measured reflections
1404 independent reflections
1176 reflections with *I* > 2σ(*I*)
*R*_int_ = 0.014

#### Refinement

*R*[*F* ^2^ > 2σ(*F* ^2^)] = 0.033
*wR*(*F* ^2^) = 0.092
*S* = 1.05
1404 reflections
109 parameters
H-atom parameters constrained
Δρ_max_ = 0.12 e Å^−3^
Δρ_min_ = −0.26 e Å^−3^

### 

Data collection: *SMART* (Bruker, 1998 ▶); cell refinement: *SAINT* (Bruker, 1998 ▶); data reduction: *SAINT*; program(s) used to solve structure: *SHELXS97* (Sheldrick, 2008 ▶); program(s) used to refine structure: *SHELXL97* (Sheldrick, 2008 ▶); molecular graphics: *SHELXTL* (Sheldrick, 2008 ▶); software used to prepare material for publication: *SHELXTL*.

## Supplementary Material

Crystal structure: contains datablock(s) I, global. DOI: 10.1107/S1600536811021210/aa2009sup1.cif

Structure factors: contains datablock(s) I. DOI: 10.1107/S1600536811021210/aa2009Isup2.hkl

Supplementary material file. DOI: 10.1107/S1600536811021210/aa2009Isup3.cml

Additional supplementary materials:  crystallographic information; 3D view; checkCIF report

## Figures and Tables

**Fig. 1.**
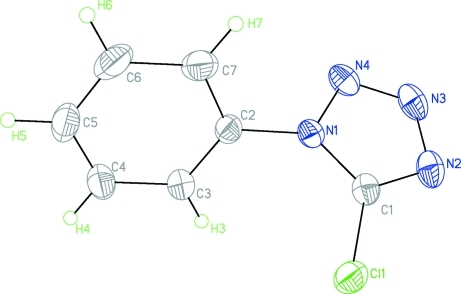
A view of (I), with displacement ellipsoids drawn at the 30% probability level. Hydrogen atoms are drawn as circles.

## supplementary crystallographic information

### Comment

The design and synthesis of new tetrazole derivatives have attracted much interest owing to their ferroecletric (Sengupta & Mukherjee, 2010), luminescent (Wang *et al.*, 2005) and magnetic properties (Grunert *et al.*, 2004; Van Koningsbruggen *et al.*, 2000). The crystal structure of 5-chloro-1-phenyl-1*H*-tetrazole (I) is shown in Fig. 1.

### Experimental

5-Chloro-1-phenyl-1*H*-tetrazole (I) (54.18 mg, 0.3 mmol) was stirred for 0.5 h in H_2_O (5 ml) and CH_3_CN (5 ml). Upon slow evaporation of the filtrate at room temperatre for two weeks, well shaped colorless crystals suitable for X-ray diffraction were obtained. Yield: 90%. Elemental analysis calcd (%) for (I): C 46.55, H 2.79, N 31.02%; found: C 46.26, H 2.68, N 31.37%.

### Refinement

H atoms were introduced in their idealized positions and refined as riding with C—H 0.93 Å.

### Crystal data

**Table:**

C_7_H_5_ClN_4_	*F*(000) = 368
*M**_r_* = 180.60	*D*_x_ = 1.519 Mg m^−^^3^
Monoclinic, *P*2_1_/*n*	Mo *K*α radiation, λ = 0.71073 Å
Hall symbol: -P 2yn	Cell parameters from 1689 reflections
*a* = 7.0428 (7) Å	θ = 3.0–27.2°
*b* = 6.4150 (6) Å	µ = 0.43 mm^−^^1^
*c* = 17.5804 (18) Å	*T* = 296 K
β = 96.160 (2)°	Block, colourless
*V* = 789.69 (13) Å^3^	0.15 × 0.14 × 0.13 mm
*Z* = 4	

### Data collection

**Table:**

Bruker SMART CCD area-detector diffractometer	1404 independent reflections
Radiation source: fine-focus sealed tube	1176 reflections with *I* > 2σ(*I*)
graphite	*R*_int_ = 0.014
φ and ω scans	θ_max_ = 25.0°, θ_min_ = 2.3°
Absorption correction: multi-scan (*SADABS*; Sheldrick, 1998)	*h* = −5→8
*T*_min_ = 0.939, *T*_max_ = 0.947	*k* = −7→7
3879 measured reflections	*l* = −20→20

### Refinement

**Table:**

Refinement on *F*^2^	Primary atom site location: structure-invariant direct methods
Least-squares matrix: full	Secondary atom site location: difference Fourier map
*R*[*F*^2^ > 2σ(*F*^2^)] = 0.033	Hydrogen site location: inferred from neighbouring sites
*wR*(*F*^2^) = 0.092	H-atom parameters constrained
*S* = 1.05	*w* = 1/[σ^2^(*F*_o_^2^) + (0.0501*P*)^2^ + 0.1635*P*] where *P* = (*F*_o_^2^ + 2*F*_c_^2^)/3
1404 reflections	(Δ/σ)_max_ < 0.001
109 parameters	Δρ_max_ = 0.12 e Å^−^^3^
0 restraints	Δρ_min_ = −0.26 e Å^−^^3^

### Special details

**Table:**

Geometry. All s.u.'s (except the s.u. in the dihedral angle between two l.s. planes) are estimated using the full covariance matrix. The cell s.u.'s are taken into account individually in the estimation of s.u.'s in distances, angles and torsion angles; correlations between s.u.'s in cell parameters are only used when they are defined by crystal symmetry. An approximate (isotropic) treatment of cell s.u.'s is used for estimating s.u.'s involving l.s. planes.
Refinement. Refinement of *F*^2^ against ALL reflections. The weighted *R*-factor *wR* and goodness of fit *S* are based on *F*^2^, conventional *R*-factors *R* are based on *F*, with *F* set to zero for negative *F*^2^. The threshold expression of *F*^2^ > σ(*F*^2^) is used only for calculating *R*-factors(gt) *etc*. and is not relevant to the choice of reflections for refinement. *R*-factors based on *F*^2^ are statistically about twice as large as those based on *F*, and *R*- factors based on ALL data will be even larger.

### Fractional atomic coordinates and isotropic or equivalent isotropic displacement parameters (Å^2^)

**Table:**

	*x*	*y*	*z*	*U*_iso_*/*U*_eq_	
Cl1	0.15030 (6)	0.26017 (7)	0.03941 (3)	0.05392 (19)	
N1	0.5232 (2)	0.25483 (17)	0.08796 (8)	0.0387 (3)	
N2	0.4612 (3)	0.24646 (19)	−0.03563 (9)	0.0527 (4)	
N3	0.6525 (3)	0.2414 (2)	−0.01533 (10)	0.0558 (4)	
N4	0.6930 (2)	0.24610 (19)	0.05813 (10)	0.0504 (4)	
C1	0.3852 (3)	0.2547 (2)	0.02910 (10)	0.0416 (4)	
C2	0.5109 (2)	0.2662 (2)	0.16905 (9)	0.0417 (4)	
C3	0.4356 (2)	0.4431 (3)	0.19789 (9)	0.0505 (4)	
H3	0.3949	0.5534	0.1659	0.061*	
C4	0.4216 (3)	0.4538 (4)	0.27567 (10)	0.0646 (6)	
H4	0.3699	0.5716	0.2963	0.078*	
C5	0.4836 (3)	0.2919 (4)	0.32218 (11)	0.0709 (7)	
H5	0.4740	0.3003	0.3744	0.085*	
C6	0.5600 (3)	0.1167 (4)	0.29252 (11)	0.0725 (6)	
H6	0.6029	0.0080	0.3249	0.087*	
C7	0.5737 (2)	0.1004 (3)	0.21435 (10)	0.0570 (5)	
H7	0.6235	−0.0183	0.1936	0.068*	

### Atomic displacement parameters (Å^2^)

**Table:**

	*U*^11^	*U*^22^	*U*^33^	*U*^12^	*U*^13^	*U*^23^
Cl1	0.0445 (3)	0.0682 (3)	0.0478 (3)	−0.00030 (19)	−0.0010 (2)	0.00150 (18)
N1	0.0380 (7)	0.0418 (7)	0.0376 (7)	0.0000 (5)	0.0103 (6)	−0.0016 (5)
N2	0.0734 (11)	0.0471 (9)	0.0396 (8)	−0.0045 (7)	0.0160 (8)	−0.0009 (6)
N3	0.0706 (11)	0.0479 (9)	0.0542 (10)	−0.0037 (7)	0.0309 (8)	−0.0039 (6)
N4	0.0477 (8)	0.0495 (8)	0.0577 (10)	−0.0012 (6)	0.0218 (7)	−0.0035 (6)
C1	0.0509 (10)	0.0367 (8)	0.0380 (9)	−0.0018 (6)	0.0083 (8)	0.0009 (6)
C2	0.0345 (8)	0.0559 (10)	0.0350 (8)	−0.0008 (7)	0.0052 (7)	0.0007 (6)
C3	0.0512 (10)	0.0596 (11)	0.0411 (9)	0.0060 (8)	0.0069 (7)	−0.0032 (8)
C4	0.0580 (12)	0.0918 (15)	0.0448 (10)	0.0055 (10)	0.0089 (9)	−0.0151 (10)
C5	0.0493 (11)	0.128 (2)	0.0349 (10)	−0.0011 (12)	0.0031 (8)	0.0015 (11)
C6	0.0518 (12)	0.1107 (18)	0.0532 (11)	0.0097 (12)	−0.0020 (9)	0.0329 (12)
C7	0.0463 (10)	0.0684 (12)	0.0567 (11)	0.0117 (8)	0.0077 (8)	0.0134 (9)

### Geometric parameters (Å, °)

**Table:**

Cl1—C1	1.6841 (18)	C3—C4	1.383 (2)
N1—C1	1.341 (2)	C3—H3	0.9300
N1—N4	1.357 (2)	C4—C5	1.364 (3)
N1—C2	1.439 (2)	C4—H4	0.9300
N2—C1	1.309 (2)	C5—C6	1.373 (3)
N2—N3	1.357 (3)	C5—H5	0.9300
N3—N4	1.293 (2)	C6—C7	1.392 (3)
C2—C3	1.373 (2)	C6—H6	0.9300
C2—C7	1.373 (2)	C7—H7	0.9300
			
C1—N1—N4	107.28 (15)	C4—C3—H3	120.7
C1—N1—C2	130.44 (14)	C5—C4—C3	120.20 (19)
N4—N1—C2	122.27 (14)	C5—C4—H4	119.9
C1—N2—N3	105.02 (16)	C3—C4—H4	119.9
N4—N3—N2	111.63 (15)	C4—C5—C6	120.57 (18)
N3—N4—N1	106.14 (16)	C4—C5—H5	119.7
N2—C1—N1	109.93 (17)	C6—C5—H5	119.7
N2—C1—Cl1	126.31 (16)	C5—C6—C7	120.52 (18)
N1—C1—Cl1	123.75 (14)	C5—C6—H6	119.7
C3—C2—C7	122.63 (16)	C7—C6—H6	119.7
C3—C2—N1	118.32 (14)	C2—C7—C6	117.56 (18)
C7—C2—N1	119.05 (14)	C2—C7—H7	121.2
C2—C3—C4	118.51 (17)	C6—C7—H7	121.2
C2—C3—H3	120.7		
			
C1—N2—N3—N4	0.03 (16)	N4—N1—C2—C3	115.00 (16)
N2—N3—N4—N1	−0.05 (16)	C1—N1—C2—C7	116.21 (18)
C1—N1—N4—N3	0.04 (15)	N4—N1—C2—C7	−65.26 (19)
C2—N1—N4—N3	−178.79 (12)	C7—C2—C3—C4	−0.4 (3)
N3—N2—C1—N1	−0.01 (15)	N1—C2—C3—C4	179.32 (15)
N3—N2—C1—Cl1	−178.96 (11)	C2—C3—C4—C5	0.7 (3)
N4—N1—C1—N2	−0.02 (16)	C3—C4—C5—C6	−0.1 (3)
C2—N1—C1—N2	178.68 (13)	C4—C5—C6—C7	−0.7 (3)
N4—N1—C1—Cl1	178.96 (10)	C3—C2—C7—C6	−0.4 (3)
C2—N1—C1—Cl1	−2.3 (2)	N1—C2—C7—C6	179.91 (16)
C1—N1—C2—C3	−63.5 (2)	C5—C6—C7—C2	0.9 (3)
